# Novel Solid Dispersions of Naphthoquinone Using Different Polymers for Improvement of Antichagasic Activity

**DOI:** 10.3390/pharmaceutics12121136

**Published:** 2020-11-24

**Authors:** Verônica da Silva Oliveira, Elen Diana Dantas, Anna Thereza de Sousa Queiroz, Johny Wysllas de Freitas Oliveira, Marcelo de Sousa da Silva, Patricia Garcia Ferreira, Fernando de Carvalho da Siva, Vitor Francisco Ferreira, Ádley Antonini Neves de Lima

**Affiliations:** 1 Department of Pharmacy, Health Sciences Center, Federal University of Rio Grande do Norte, Natal, Rio Grande do Norte 59012-570, Brazil; veronicasoliver47@gmail.com (V.d.S.O.); elendiana88@gmail.com (E.D.D.); anynhathereza@ufrn.edu.br (A.T.d.S.Q.); johnywysllas@gmail.com (J.W.d.F.O.); mssilva.ufrn@gmail.com (M.d.S.d.S.); 2 Global Health and Tropical Medicine, Institute of Hygiene and Tropical Medicine, NOVA University Lisbon, 1800-166 Lisbon, Portugal; 3 Department of Pharmaceutical Technology, Faculty of Pharmacy, Federal Fluminense University, Niterói, Rio de Janeiro 24241-002, Brazil; patricia.pharma@yahoo.com.br (P.G.F.); vitorferreira@id.uff.br (V.F.F.); 4 Institute of Chemistry, Federal University Fluminense, Niterói, Rio de Janeiro 24020-150, Brazil; fcsilva@id.uff.br

**Keywords:** solid dispersion, delivery system, polymeric carriers, antichagasic activity

## Abstract

IVS320 (3a,10b-dihydro-1*H*-cyclopenta[*b*]naphtho[2,3-*d*]furan-5,10-dione) is a naphthoquinone that has low solubility in aqueous medium, a physical behavior that limits its biological activities, considering that compounds from this class have several activities. In this work, solid dispersions (SDs) prepared between IVS320 and polymers hydroxypropyl methylcellulose (HPMC), polyethylene glycol (PEG), and polyvinylpyrrolidone (PVP) were developed using physical mixture (PM), kneading (KN), and rotary evaporation (RE) methods. Dispersions were investigated using Fourier transform infrared spectroscopy (FTIR), differential scanning calorimetry (DSC), thermogravimetry (TG), powder X-ray diffraction (PXRD), and scanning electron microscopy (SEM). In addition, in vitro antiparasitic activity in *Trypanosoma cruzi* Y strains was evaluated. Physical-chemical characterization demonstrated the formation of SDs through the interaction of IVS320 with polymeric matrices. SDs of IVS320-polymer presented a significant potentiation of antichagasic activity, with inhibitory growth around 62% (IVS320-HPMC/RE), 55% (IVS320-PEG/RE), and 85% (IVS320-PVP/RE), while pure IVS320 showed a value of 48% for the highest concentrations evaluated (50 µg/mL).

## 1. Introduction

The parasitic infection caused by *Trypanosoma cruzi*, known as Chagas disease, is included among a list of 20 neglected tropical diseases, affecting around 6 to 8 million people and killing about 15 thousand per year [1]. The main form of transmission requires a vector, with *Triatoma infestans* being the most common insect [2,3]; this type of transmission corresponds to approximately 80% of infections. In addition to this route, *T. cruzi* can be transmitted by blood transfusion, transplant of solid organs, congenital infection, inoculation by accident at work, or orally, through the ingestion of contaminated substances [1].

**Figure 1:**
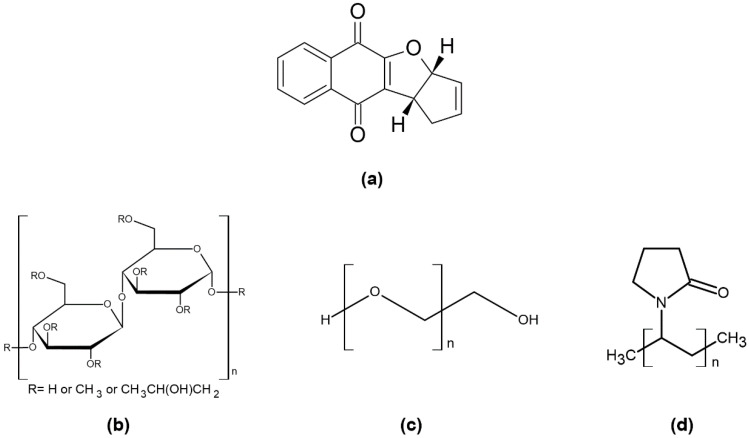
Chemical structure: (**a**) 3a,10b-dihydro-1*H*-cyclopenta[*b*]naphtho[2,3-*d*]furan-5,10-dione (IVS320), (**b**) hydroxypropyl methylcellulose (HPMC), (**c**) polyethylene glycol (PEG), and (**d**) polyvinylpyrrolidone (PVP).

In recent years, the interest in studying naphthoquinones has intensified, both for its relevance in vital biochemical processes, as well as for its variety of known pharmacological properties, such as anti-inflammatory [4,5], antitumor [6,7], antibacterial, antifungal [8], antiviral [7,9,10], antimalarial [11], leishmanicidal [12], and trypanocidal activities [13,14,15]. Among the naphthoquinone compounds included in the quinone family, there is the IVS320 molecule (Figure 1a), chemically named 3a,10b-dihydro-1*H*-cyclopenta[*b*]naphtho[2,3-*d*]furan-5,10-dione, produced synthetically and with proven antifungal [16] and antichagasic activity [17].

However, IVS320, like most new drug candidates, presents low solubility in aqueous medium [17], causing an impact on the dissolution rate and, consequently, on its bioavailability. There are several ways to get around this problem of solubility and stability, such as inclusion complexes [18] and solid dispersion systems.

In this case, for the elaboration of solid dispersions (SDs), polymers play a prominent role, as they can stabilize SDs due to their ability to retain the drug in an amorphous form in the polymeric matrix during storage [19], and can increase the wettability, dispersibility, and solubility of drugs [20].

Some examples of these polymers are polyvinylpyrrolidone (PVP) [21], polyethylene glycol (PEG), and various cellulose derivatives, such as hydroxypropyl methylcellulose (HPMC) [22,23], which are hydrophilic polymer carriers used as excipients in several pharmaceutical formulations, due to (among other characteristics) their low toxicity [24,25,26].

In view of the applications of naphthoquinones, pre-preliminary studies of the biological activities of compound IVS320, and the need to develop alternatives for the treatment of Chagas disease, this work aimed to use solutions centered on pharmaceutical technology to improve the physicochemical and biological properties of naphthoquinone IVS320, through the preparation of SDs in polymeric matrices.

In this article, SDs of IVS320 (Figure 1a) were prepared by kneading and rotary evaporation methods with polymers to improve the antichagasic activity of IVS320. Three polymers were selected as carriers: hydroxypropyl methylcellulose (HPMC) (Figure 1b), polyethylene glycol (PEG) (Figure 1c), and polyvinylpyrrolidone (PVP) (Figure 1d).

## 2. Materials and Methods

### 2.1. Material

The compound IVS320 (3a,10b-dihydro-1*H*-cyclopenta[*b*]naphtho[2,3-*d*]furan-5,10-dione) is a dihydrofuran fused 1,4-naphthoquinone that was synthesized by the LabSOA (Laboratory of Applied Organic Synthesis) at the Federal University Fluminense [17]. The polymers hydroxypropyl methylcellulose (HPMC), polyethylene glycol 6000 (PEG), and polyvinylpyrrolidone K-30 (PVP) were purchased from Sigma Aldrich Corporation (San Luis, MO, USA), and the solvents used were all analytical grade.

### 2.2. Preparation of the SDs

Solid dispersions (SDs) were prepared via physical mixture (PM), kneading (KN), and rotary evaporation (RE) methods using the ratio (1:1, *w*/*w*) IVS320:polymer for all methodologies.

The preparation of the physical mixture (PM) of IVS320 and the respective polymer (HPMC, PEG, and PVP) were carried out in a (1:1) ratio, subsequently homogenized with mortar and pestle, and then stored in the dissector.

In the kneading method (KN), the IVS320 and each polymer were weighed, homogenized with mortar and pestle, and then addition a mixture of water/acetone solvent (50:50, *v*/*v*) was added. Afterwards, this solution was dried in an oven at 60 °C for 24 h, and the solid obtained was stored in a desiccator.

The rotary evaporation method (RE) consisted of solubilizing the IVS320 and the polymers in a mixture of water/acetone solvents (50:50, *v*/*v*). Then, this solution was subjected to constant stirring at 25 °C for 72 h. Finally, the solution was evaporated under vacuum at 75 °C, and the solid obtained was stored in a desiccator.

### 2.3. Characterization of the SDs

#### 2.3.1. Fourier Transform Infrared Spectroscopy (FTIR)

Infrared spectroscopic analysis was performed using IR Prestige-21 equipment (Shimadzu Corporation, Kyoto, Japan). The analysis was carried out in the 4000–600 cm^−1^ region with 15 scans.

#### 2.3.2. Thermal Analysis

Differential scanning calorimetry (DSC) thermal analysis was carried out in a DSC-50 cell Shimadzu^®^ (Tokyo, Japan) using approximately 2 mg of sample in aluminum crucibles under dynamic nitrogen atmosphere (50 mL·min^−1^) and with a heating rate of 10 °C·min^−1^ at a temperature range of 30–500 °C.

Thermogravimetry (TG) was obtained using a TGA-50 Shimadzu^®^ (Tokyo, Japan) in the temperature range of 30–900 °C, using alumina crucibles with approximately 2 mg of samples under dynamic nitrogen atmosphere (50 mL·min^−1^) and with a heating rate of 10 °C·min^−1^.

#### 2.3.3. Powder X-ray Diffraction (PXRD)

PXRD analysis was carried out using a Bruker D2 Phaser (Bruker Corporation, Billerica, MA, USA) with CuKα radiation (λ = 1.54 Å) at a voltage of 30 kV and a current of 15 mA, using a Lynxeye detector (Bruker Corporation, Billerica, MA, USA). Samples were scanned at room temperature for 2 h at a range of 5–40° at 0.05° s^−1^.

#### 2.3.4. Scanning Electronic Microscopy (SEM)

Samples were mounted on aluminum stubs using double-sided adhesive tape. Morphological analysis was performed on a Hitachi TM-3000 Tabletop Microscope (Hitachi Ltd., Tokyo, Japan) at a magnification of 500×. SEM images were obtained at an accelerating potential of 15 kV under reduced pressure.

### 2.4. Antichagasic Activity

#### 2.4.1. Parasite

The *T. cruzi* Y strain was grown in LIT (liver infusion tryptose) medium supplemented with 10% SFB (serum fetal bovine) and 5% streptococcus/penicillin antibiotic (100 UI/mL), and kept at 27 °C in a BOD (Biochemical Oxygen Demand) oven to obtain the epimastigote forms of the parasite.

#### 2.4.2. In Vitro Antichagasic Evaluation

The parasites were diluted and counted with the aid of a Neubauer improved camera (New optics, El Centro, CA, USA) and used at a concentration of 1 × 10^7^ parasites/mL. The IVS320 compound and the IVS320-polymer systems were solubilized in dimethylsulfoxide (DMSO) with a maximum concentration of 1% in 96-well plates. Briefly, a stock solution was prepared for each of the evaluated systems, and subsequently diluted in the culture medium, obtaining concentrations in the range of 50 µg/mL to 2.5 µg/mL for the pure IVS320 and the concentration of the IVS320 in the SDs. Then cultures of the epimastigote form of *T. cruzi* were dispensed in 96-well plates and incubated for 24 h. The positive control contained only medium and strain; negative controls (medium and drug) and solvent control (contained 1% DMSO, medium and strain) were also obtained to ensure that the solvent did not influence the assay. The inhibition of *T. cruzi* was evaluated by resazurin reduction assay (Sigma Aldrich). After 24 h, a reading was performed at 570 nm and 600 nm using a microplate reader (Epoch, BioTek Instruments, Winooski, VT, USA). Experiments were performed in triplicate, and benznidazole was used as a trypanocidal reference drug. The inhibition percentage was based on the following formula:

% Inhibition = 100 − ((A_570t_ − (A_600t_ × R_0_))/(A_570c_ − (A_600c_ × R_0_))) × 100 (1)

A_570t_ = Absorption of the treatment at 570 nm wavelength.

A_600t_ = Absorption of the treatment at 600 nm wavelength.

A_570c_ = Absorption of control at 570 nm wavelength.

A_600c_ = Absorption of the control at 600 nm wavelength.

R_0_ = Medium correction factor interacting with resazurin.

R_0_ = Am_570_/Am_600_ (2)

Am_570_ = Absorbance of the medium at a wavelength of 570 nm.

Am_600_ = Absorbance of the medium at a wavelength of 600 nm.

#### 2.4.3. Statistical Analysis

Data analysis was performed using GraphPad Prism software version 5.0 (La Jolla, CA, USA). The results are presented as mean ± standard error of the mean (S.E.M.). A one-way analysis of variance (ANOVA) was performed to determine the significant differences between the groups, followed by Dunnett’s *t*-test for multiple comparisons. Values of *p* < 0.05 were considered significant.

## 3. Results and Discussion

### 3.1. Fourier Transform Infrared Spectroscopy (FTIR)

The infrared spectrum of IVS320 showed vibrational modes referring to νC=O asymmetrical and symmetrical, respectively, at 1678 and 1644 cm^−1^, characteristic of the carbonyl group, corroborating with the stretching of 1,4-naphthoquinone, lawsone, and lapachol [27,28,29], as well as bands at 1614, 1591, and 1568 cm^−1^, attributed to C=C stretching vibration present in the benzene ring and cyclopentene in the structure of the IVS320. Other bands were observed in the region from 1300 to 1020 cm^−1^, corresponding to C-O-C and C-O (aromatic ether) stretching.

Analyzing the polymers spectra, HPMC exhibited bands at 3457 cm^−1^ attributed to the O-H stretching and at 1053 cm^−1^ corresponding to C-O-C stretching [20]. In the PEG, a broad band at 3477 cm^−1^ (νO-H) and a peak at 1096 cm^−1^ (νC-O) were observed, characteristic of ether. The PVP exhibited a broad band in the region of 3412 cm^−1^, referring to the presence of water and corroborating with the DSC results of the polymer. Additionally, bands were also observed for PVP at 2933 cm^−1^, 1648 cm^−1^, and 1287 cm^−1^, which were attributed to C-H, C=O (pyrrolidone group), and C-N stretching, respectively [30].

The SDs exhibited vibrational modes characteristic of the IVS320 molecule and isolated polymers, as well as a shift in the stretching vibration and changes in the profiles of some bands. Some changes were observed in the range of 1400–1170 cm^−1^, corresponding to the vibrational modes of various C-C, C-O, and C-O-C bonds, and C-H and O-H deformations.

**Figure 2:**
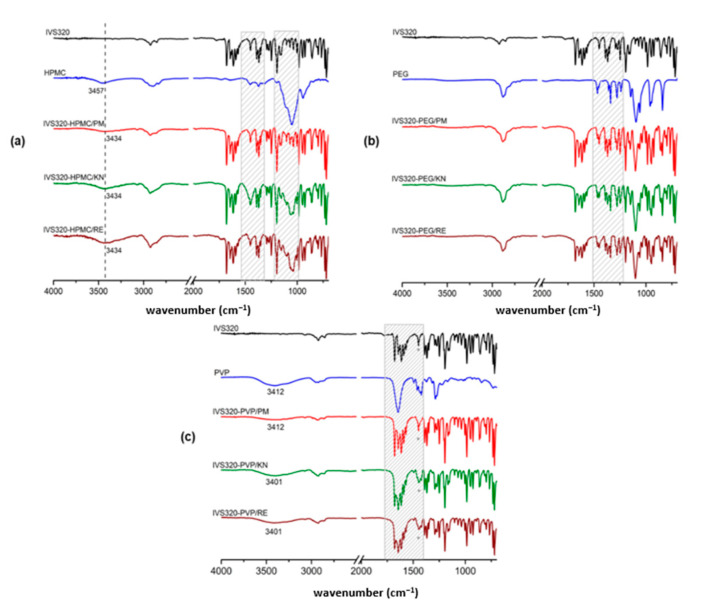
Infrared spectrum of systems: (**a**) IVS320-HPMC, (**b**) IVS320-PEG, and (**c**) IVS320-PVP.

In the spectrum of SDs obtained with HPMC (Figure 2a), the presence of νO-H and νC-H was found to correspond with HPMC. In addition to a 1053 cm^−1^ band referring to C-O-C stretching, in the region from 1115 to 1016 cm^−1^ in the same spectrum, changes were observed in the profile of the three bands corresponding to the IVS320, located at 1102, 1069, and 1035 cm^−1^, due to interactions between the IVS320 and the polymer, suggesting the formation of dispersions. By analyzing the spectra (Figure 2a), the physical mixture proved to be less effective at showing the IVS320-HPMC interaction, considering that the characteristic bands of the polymer were not very evident.

IVS320-PEG SDs showed changes in the profile of the bands in the range of 1510 to 1213 cm^−1^. In IVS320-PEG/KN and IVS320-PEG/RE SDs, the band displayed at 1248 cm^−1^ corresponding to the IVS320 underwent a small shift to 1250 cm^−1^ (C-O stretching). The presence of PEG in the dispersions was evidenced by the vibrational modes corresponding to this polymer, with small variations. In the SDs, bands were found at 1102 cm^−1^ referring to C-O deformation characteristic of ether, while in the PEG, it was at 1096 cm^−1^.

In the range of 1678 to 1407 cm^−1^ in the spectrum of the IVS320-PVP SDs (Figure 2c), modifications were observed in the ratio of the intensity of the bands referring to C=O and C=C stretching, showing that such changes may be due to interactions between the IVS320 molecule and the polymeric chain through these functional groups, thus changing the profile of these bands. While in the spectrum of the physical mixture (IVS320-PVP/PM), the profile of this set of bands remained practically unchanged. Additionally, in the dispersions, it was possible to verify the O-H stretching referring to the presence of the polymer, showing small displacements when compared to the isolated polymer, with a value of 3412 cm^−1^, and 3401 cm^−1^ in the PVP.

IVS320-HPMC SDs exhibited peak shifts for the O-H stretching (from 3457 cm^−1^ to 3434 cm^−1^), C-O stretching (from 1248 cm^−1^ to 1250 cm^−1^), and C-O-C stretching (from 1194 cm^−1^ to 1197 cm^−1^). Similar shifts were observed for IVS320-PEG SDs (Figure 2b). In addition, in the IVS320-PEG SDs, a shift was observed regarding the C-O stretching of the PEG (from 1096 to 1102 cm^−1^). These changes suggest the possibility of hydrogen bonding between the hydroxyl of HPMC and the carbonyl group of IVS320.

Hydrogen bonds between the carbonyl group (C=O) of the IVS320 and the hydroxyl of the HPMC or PEG polymers would be expected. However, spectral changes were observed, mainly in relation to the O-H, C-O, and C-O-C groups, which may suggest that interactions occur between the ether group of the IVS320 and the hydroxyl of the polymers. On the other hand, in the spectrum of the IVS320-PVP system, the main changes were found in the profile of the bands corresponding to C=O and C=C stretching, possibly attributed to the interactions between the C=O group of the IVS320 and polymer.

### 3.2. Differential Scanning Calorimetry (DSC)

**Figure 3:**
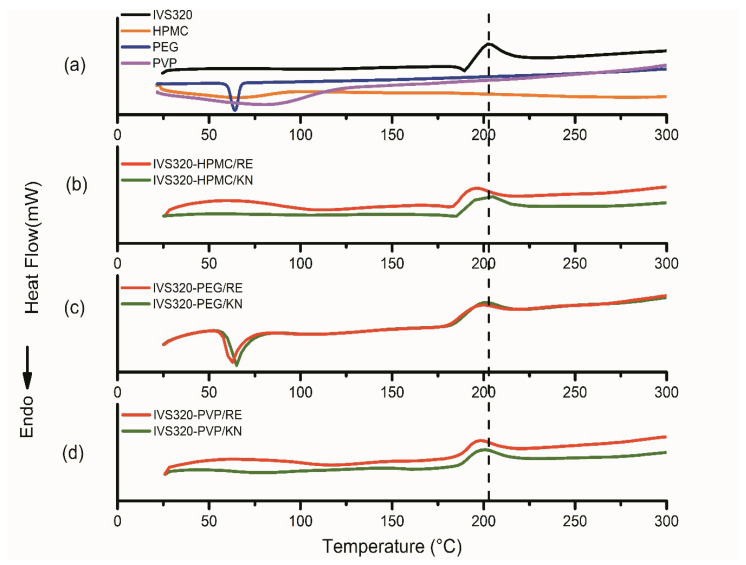
Differential scanning calorimetry (DSC) curve of (**a**) IVS320 and polymers, (**b**) solid dispersions (SDs) of IVS320-HPMC, (**c**) SDs of IVS320-PEG, and (**d**) SDs of IVS320-PVP. RE: rotary evaporation; KN: kneading.

The DSC curve of the IVS320 showed two events (Figure 3a): the first, an endothermic event peaking at 189 °C, corresponding to its melting point; the second, an exothermic event at 203 °C, attributed to possible changes in the crystalline form of the IVS320.

The thermogram of the HPMC and PVP polymers (Figure 3a) showed a broad endothermic peak, as a result of dehydration, in the range of 75 to 135 °C [30], while no peak corresponding to the melting point was found for these polymers, considering their amorphous structure [20]. However, PEG revealed an endothermic peak at a temperature around 63.9 °C, corresponding to its melting point, according to literature reports [20,31].

In the IVS320-HPMC/KN SD, an endothermic event at 184 °C and another exothermic at 200 °C were observed, both attributed to the IVS320 present in the polymeric matrix, while in IVS320-HPMC/RE SD, these same events were recorded at 181 °C and 195 °C. In the DSC thermograms (Figure 3b), characteristic fusion peaks of the IVS320 still existed, indicating the microcrystalline form of the IVS320 in the HPMC matrix, according to results reported in the 1:1 ratio (drug:polymer) [32].

In the DSC curve of the IVS320-PEG system, the process related to the melting point of the PEG showed variations that were observed at 65.2 °C in IVS320-PEG/KN SD and 62.7 °C in IVS320-PEG/RE SD. The endothermic events related to the IVS320 were not observed, while the exothermic events were observed with temperature variations, with values of 200 °C (IVS320-PEG/KN) and 198 °C (IVS320-PEG/RE) for SDs (Figure 3c).

There was also no endothermic event related to IVS320 in the IVS320-PVP SDs (Figure 3d); this indicates that the IVS320 was converted into an amorphous state, thus justifying its dispersion in the polymeric carrier. However, the exothermic event was found at 200 °C in the IVS320-PVP/KN SD and at 198 °C in the IVS320-PVP/RE SD [33].

Analyzing DSC data, it was found that in all dispersions, the exothermic event referring to the IVS320 underwent small temperature variations when compared to the isolated IVS320, implying that such changes occurred due to possible modifications in the crystalline form of the IVS320, thus suggesting its interaction in the polymeric matrices.

### 3.3. Thermogravimetric Analysis (TG)

**Figure 4:**
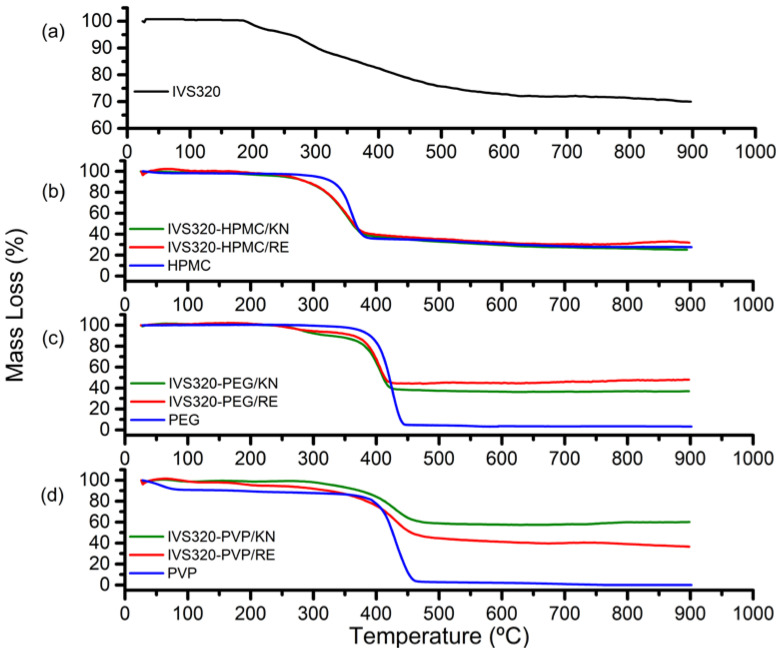
TG curves of (**a**) IVS320, (**b**) SDs of IVS320-HPMC, (**c**) SDs of IVS320-PEG, and (**d**) SDs of IVS320-PVP under N_2_ atmosphere.

The TG curve of the IVS320 shows three mass loss events (Figure 4a). The first occurred in the range of 150 to 162 °C, with approximately 5% mass loss. The second variation occurred in the temperature range from 262 to 315 °C, with about 6.25% mass loss. Lastly, the third event was found in the range of 315 to 900 °C, with a reduction of 18.73%, as reported by Dantas et al. [17]. This high thermal resistance of the IVS320 is consistent with some substances of the naphthoquinone class [34].

The TG curve of the HPMC exhibited a degradation process, in the temperature range of 250 to 395 °C (peak 355 °C), with about 61% mass loss (Δm). The SDs formed between the IVS320 and the HPMC exhibited, in the range 210 to 390 °C (peak 345 °C), a mass loss of 58.12% and 57.41% for the dispersions IVS320-HPMC/KN and IVS320-HPMC/RE, respectively. In the range of 390 to 700 °C, a gradual mass loss of around 10.9% (IVS320-HPMC/KN) and 9.81% (IVS320-HPMC/RE) was observed, while in the TG curve of HPMC, a mass loss of around 7.41% was observed (Figure 4b).

The PEG polymer showed major mass loss, with a mass loss of approximately 95.3% in the range of 290 to 450 °C (Figure 4c). The IVS320-PEG SDs showed variations in mass loss at temperatures from 220 to 315 °C, with mass losses of 9.27% (IVS320-PEG/KN) and 6.36% (IVS320-PEG/RE). On the other hand, in the range of 315 to 440 °C, higher mass losses were observed, with values of 52.3% for the SD of IVS320-PEG/KN and about 49.17% for the SD of IVS320-PEG/RE.

The TG curve of the PVP exhibited two mass losses: the first, in the range of 30 to 100 °C, with a mass variation of 8.77%, corresponding to the presence of water; the second, in the range of 357 to 480 °C, with a mass loss of 83.16%, attributed to the process of thermal polymer degradation, corroborating the literature [35,36]. For SDs with PVP, in the range of 137 to 245 °C, mass variations of 3.53% (IVS320-PVP/RE) and 1% (IVS320-PVP/KN) were found. In turn, in the range of 245 to 480 °C, mass losses of 49.26% and 39.75% were obtained, respectively, for the SDs of IVS320-PVP/RE and IVS320-PVP/KN (Figure 4d).

Analyzing the percentages of mass loss among all dispersions, those obtained with the PVP polymer presented the smallest variations in mass, mainly the IVS320-PVP/KN SD, suggesting that at higher temperatures, there was an increase in the thermal stability of the dispersion.

According to the TG and DSC curves of the IVS320-polymer systems, the materials obtained have a stable thermal profile, showing that the presence of IVS320 does not interfere with the thermal profile of the polymeric carriers, which is behavior that strongly suggests a good interface between compound and polymers [37].

### 3.4. Powder X-ray Diffraction (PXRD)

The X-ray diffractograms of IVS320 presented high intensity peaks at 10.50°, 14.34°, 24.38°, and 28.22°, followed by a series of secondary reflections, indicating a crystalline profile, as already reported by Dantas et al. [17].

**Figure 5:**
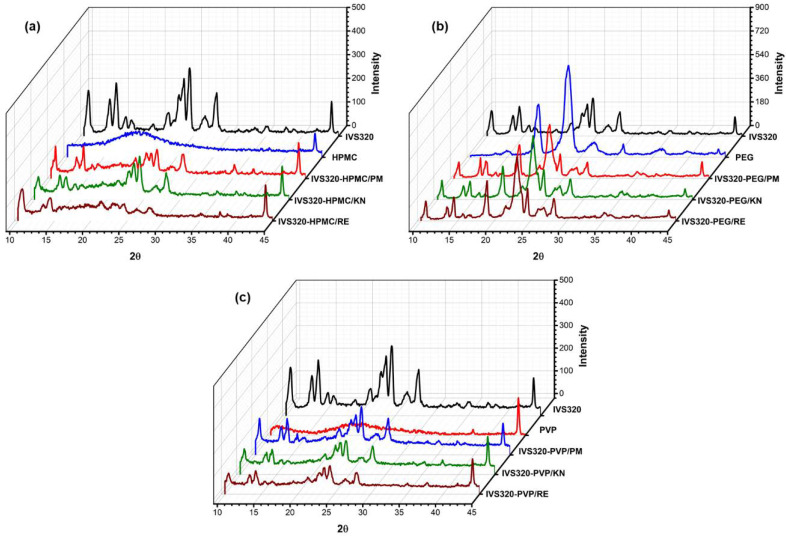
Powder X-ray diffraction (PXRD) of systems (**a**) IVS320-HPMC, (**b**) IVS320-PEG, and (**c**) IVS320-PVP.

The PEG showed two intense crystalline reflections at 19.30° and 23.43° (Figure 5b), while HPMC and PVP showed an amorphous pattern, with no peak in the diffractogram [30,35].

For all IVS320-polymer systems, a reduction in the intensity of the standard peaks was observed, indicating a change in the amorphous state, both in the dispersions obtained by the kneading (KN) and rotary evaporation (RE) methods. Evidence of less intense peaks in polymeric systems may indicate the achievement of partially amorphous shapes due to the influence of polymers.

The physical mixtures showed peaks of the individual species, showing IVS320 crystallinity characteristics with a decrease in its intensity, which is attributed to the dilution and surface coverage of the IVS320 particles by the polymers [20]. On the other hand, the PXRD profile of the SDs showed a more marked reduction in the peaks, suggesting the formation of amorphous solid dispersions.

In SDs with HPMC, a more pronounced reduction in peaks was observed, particularly in the range of 20° to 30° (Figure 5a), indicating a change in the amorphous characteristic state of pure HPMC, thus suggesting that the polymer inhibits IVS320 crystallization, mainly in the SD of IVS320-HPMC/RE.

On the other hand, the SDs obtained with PEG were more crystalline than the IVS320-HPMC and IVS320-PVP (Figure 5c), possibly suggesting a recrystallization of the IVS320 during the process of preparing its dispersions, after evaporation of the solvent. This can be confirmed by the small displacements of the peaks corresponding to the PEG, bearing in mind that in the diffractogram, in relation to the polymer, crystalline reflections appeared at 19.30° and 23.43°, while in the SDs they were found at 18.95° and 23.07°.

### 3.5. Scanning Electron Microscopy (SEM)

**Figure 6:**
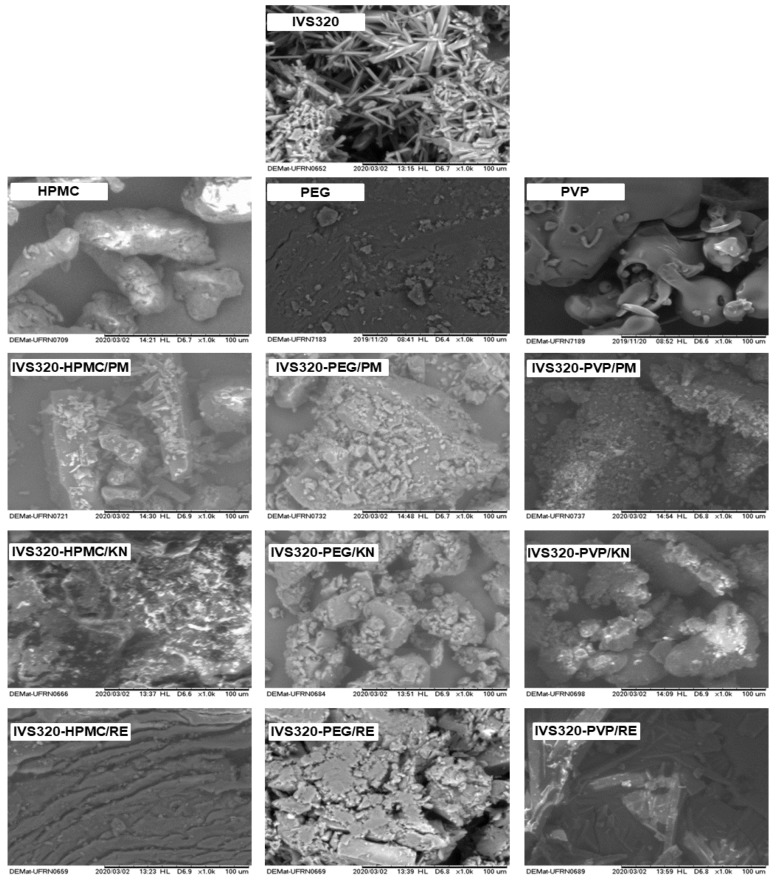
Scanning electron microscopy of IVS320, polymers (HPMC, PEG, and PVP), physical mixtures (PMs), and SDs obtained by kneading (KN) and rotary evaporation (RE).

The micrograph of the IVS320 displays very fine crystals with varying sizes and a predominance of the pyramidal shape and some crystals in the form of needles (Figure 6), corroborating the PXRD results.

HPMC exhibited an irregular cylindrical filament structure, while PEG exhibited a block-shaped structure, and the PVP particles showed irregular shapes with a smooth surface [20,30].

Images obtained for the IVS320-polymer systems by the physical mixture (PM) method showed the deposition of fragments of the IVS320 on the polymeric surface of the carriers, not verifying a uniform structure or surface that proves the interaction of the IVS320 in the polymeric network.

On the other hand, the micrographs for the IVS320-HPMC, IVS320-PEG and IVS320-PVP systems that were obtained through the kneading (KN) and rotary evaporation (RE) methods presented particles with irregular shapes and morphologies that were different from both components that originated it.

Images of the SDs of IVS320-HPMC/KN and IVS320-HPMC/RE showed rough surfaces, while the SDs of IVS320-PEG/KN and IVS320-PVP/KN exhibited images with amorphous structures of agglomerates. The IVS320-PEG/RE and IVS320-PVP/RE images suggest an association of the IVS320 with the polymer, composing a uniform matrix.

### 3.6. In Vitro Antichagasic Activity

**Figure 7:**
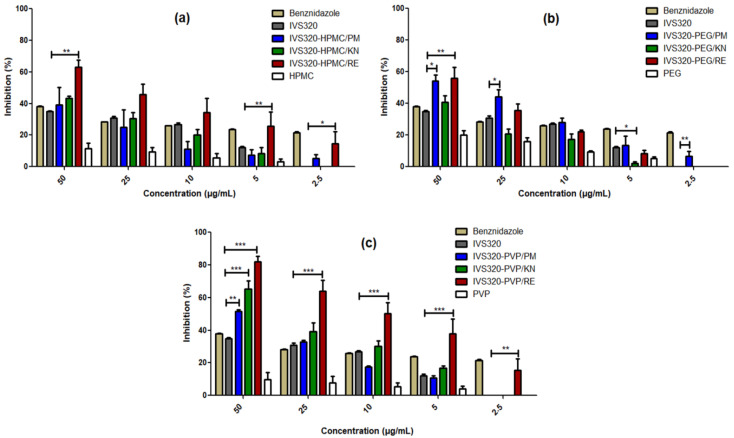
In vitro antichagasic activity of IVS320-polymer systems: (**a**) IVS320-HPMC, (**b**) IVS320-PEG, and (**c**) IVS320-PVP against epimastigotes of *T. cruzi* Y strain for 24 h. Values refer to % of control (untreated). *** *p* < 0.0001, ** *p* < 0.001 and * *p* < 0.05 compared with pure IVS320 at the same concentration. Statistical analysis was performed using ANOVA followed by Dunnett’s *t*-test for multiple comparisons.

The antichagasic activity of the pure IVS320 is practically comparable to benznidazole (reference drug) at concentrations of 50 to 10 µg/mL, as shown in Figure 7. However, the IVS320-polymer SDs showed better activity, with a higher percentage of inhibitory growth (%) when compared to the pure IVS320, which presented a value of 37% for the highest concentration evaluated (50 µg/mL).

Figure 7 reports the inhibitory growth (%) as a function of the concentration of pure IVS320 and its equivalent concentrations in the dispersions.

The IVS320-HPMC/RE dispersion showed an activity higher to pure IVS320 for all concentrations assessed in the range of 50 to 2.5 µg/mL, presenting an inhibitory growth rate around 62% for the highest concentration.

Analyzing the IVS320-PEG system at concentrations of 50 and 25 µg/mL, the IVS320-PEG/PM and IVS320-PEG/RE dispersions exhibited a greater inhibitory effect, when compared to pure IVS320 at the same concentrations, showing inhibitory activity around approximately 55% for a concentration of 50 µg/mL.

Similar to the IVS320-HPMC system, the results obtained for the IVS320-PVP system showed a better performance of the inhibitory activity for the SD of IVS320-PVP/RE, with an inhibitory rate around 82% and 64%, respectively, for the concentrations of 50 and 25 µg/mL. Additionally, it exhibited inhibitory activity higher than pure IVS320, even at the lowest concentrations, showing values of 15% inhibitory growth at the concentration of 2.5 µg/mL of IVS320 when incorporated in the SDs.

The SDs of the IVS320 with the HPMC and PVP polymers presented the best results for antichagasic activity when obtained by the rotary evaporation (RE) method. However, among the results, the IVS320-PVP/RE system stood out due to the 82% antichagasic activity rate and the improvement in inhibitory activity in relation to all the concentrations evaluated, when compared to pure IVS320. The SD of IVS320-PVP/KN also displays significant inhibitory activity, showing a rate of around 65% at a 50 µg/mL concentration.

The activity of the IVS320 incorporated in the dispersions was increased when compared to the pure IVS320, which can probably be explained as being due to the increment in the solubility of the compound in these polymeric matrices. Also, linked to this behavior, it can have the influence of the polymer. Despite the low inhibitory activity found in each of the polymers, even at higher concentrations, there may be a synergism of the inhibitory effect between the two constituents of the polymeric matrix (IVS320 and polymer), and it may also be another factor that explains the increase in the antichagasic activity of the SDs in relation to pure IVS320.

Studies carried out by Lima et al. [38] show that a reduction in particle size, an increase in surface area, and the interaction between polymeric carriers can result in the increase in the solubility of drugs.

## 4. Conclusions

Using different polymeric materials, it was possible to evaluate the interactions of carriers with the compound IVS320 to obtain SDs. The results showed changes in vibrational modes, as well as alterations in the thermal behavior and IVS320 crystallinity pattern, which in turn, led to an improvement of the IVS320 antichagasic activity, when present in polymeric matrices. The results indicated that the inhibitory growth (%) improved gradually with the increase in the concentration of the samples, and IVS320-polymer SDs showed potentiated activity in comparison with IVS320 in some concentrations. It was observed that the SD obtained by the rotary evaporation method with the PVP polymer resulted in the best antichagasic activity of the IVS320 incorporated in the SDs. This behavior shows that the addition of polymers alters the effectiveness of IVS320 in terms of its antichagasic activity, probably due to the improved solubility and bioavailability of IVS320.
